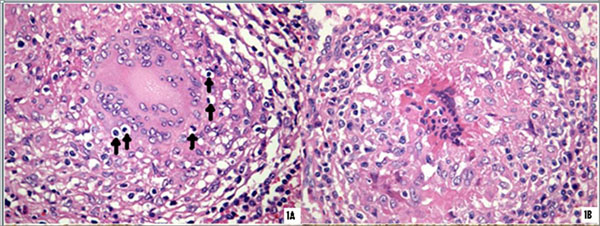# Emperipolesis and cell death in NOD2-related Blau Syndrome and Crohn’s disease

**DOI:** 10.1186/1546-0096-9-S1-P293

**Published:** 2011-09-14

**Authors:** Carl EI Janssen, Carlos D Rose, Antonio Naranjo, Brigitte Bader-Meunier, Rolando Cimaz, Miroslav Harjacek, Pierre Quartier, Rebecca TenCate, Caroline Thomee, Isabelle Cleynen, Tammy M Martin, Gert De Hertogh, Tania Roskams, Valeer J Desmet, Carine H Wouters

**Affiliations:** 1 Leuven Univesity Hospital, Belgium; 2 DuPont Children’s Hospital, Wilmington, US; 3 Hospital Universitario de Gran Canaria, Spain; 4 Hôpital Necker, Paris, France; 5 AOU Meyer and University of Florence, Italy; 6 University Hospital Zagreb, Croatia; 7 Leiden University Medical Center, The Netherlands; 8 Centre Hospitalier Luxembourg; 9 Casey Eye Institute, Portland, Oregon

## Background

Blau Syndrome (BS), a rare autoinflammatory disease characterized by non-caseating granulomas, is caused by gain-of-function mutations in NOD2. Crohn’s disease (CD) is associated with intestinal granulomas, and SNPs in NOD2. Emperipolesis, the ‘inside round about wandering’ of lymphocytes within other cells is a typical feature of Rosai-Dorfman disease, and seen occasionally in malignancies. Cell survival and cell death are possible outcomes for both the engulfed and engulfing cells.

## Aim

To investigate emperipolesis and cell death in BS and CD granulomas.

## Methods

Morphological and immunohistochemical study of granulomas was undertaken in 8 BS and 7 pediatric CD biopsies, using H&E and immunohistochemistry for leukocyte markers (CD68, CD4, CD8, CD20), cytokines (IFNγ, IL6, IL10, IL17, TGFβ, TNFα) and death-proteins (Bcl2, Fas, FasL, activated caspase 3).

## Results

All BS biopsies showed polycyclic granulomas with large lymphocytic coronas and extensive emperipolesis of lymphocytes within multinucleated giant cells (MGCs). This was associated with macrovesicular/microvesicular degeneration of lymphocytes inside MGCs (Fig1a), and MGC death (Fig1b). Emperipolesis selectively involved CD4+ T cells. In addition, vesicles and degenerative remnants inside MGCs stained strongly for IL-6 and IL-17. A moderate expression of Bcl2 was present, Fas and FasL expression were seen in emperipoletic lymphocytes and MGCs but caspase 3 was virtually absent. In contrast, CD biopsies demonstrated simple isolated granulomas with subtle lymphocytic coronas; emperipolesis was sporadically found in a few biopsies, and was associated with crystalline inclusions, but not with MGC death.

## Conclusion

Emperipolesis of CD4+lymphocytes is an important feature of BS and is associated with MGC death. NOD2 mutations causing NF-*κ*B hyperactivation and influencing autophagy pathways may be involved. In CD with NOD2-SNPs, emperipolesis is exceptional and crystalline inclusions are present. (Figure 1)

**Figure 1:**